# Hypothesis of a CD137/Eomes activating axis for effector T cells in HPV oropharyngeal cancers

**DOI:** 10.1186/s10020-024-00796-w

**Published:** 2024-02-14

**Authors:** Robin Baudouin, Eric Tartour, Cécile Badoual, Stéphane Hans

**Affiliations:** 1 Department of Otolaryngology-Head & Neck Surgery, Foch Hospital, 40 rue Worth, 92 150 Suresnes, France; 2 School of Medicine, UFR Simone Veil, Université Versailles Saint-Quentin-en- Yvelines (Paris Saclay University), 2 Av. de la Source de la Bièvre, Montigny- le-Bretonneux, 78 180 France; 3 Université Paris Cite, INSERM, PARCC, Hôpital européen Georges Pompidou, Service d’Immunologie biologique, 20, Rue Leblanc, Paris, 75015 France; 4 Hôpital européen Georges Pompidou, Service d’anatomopathologie, 20, Rue Leblanc, Paris, 75015 France

**Keywords:** Cancer, HPV, Squamous cell carcinoma, T regulator, FoxP3+, Eomes, Lymphocytes

## Abstract

Chronic Human Papilloma Virus (HPV) infection is supplanting alcohol and tobacco intoxications as the leading cause of oropharyngeal cancer in developed countries. HPV-related squamous cell carcinomas of the oropharynx (HPV + OSC) present better survival and respond better to radiotherapy and chemotherapy. Regulatory T cells (T_REG_) are mainly described as immunosuppressive and protumoral in most solid cancers. However, T_REG_ are paradoxically associated with a better prognosis in HPV + OSCs. The transcription factor FoxP3 is the basis for the identification of T_REG_. Among CD4 + FoxP3 + T cells, some have effector functions. A medical hypothesis is formulated here: the existence of a CD137 (4.1BB)-Eomesodermin (Eomes) activated pathway downstream of TCR-specific activation in a subpopulation of CD4 + FoxP3 + T cells may explain this effector function. Evidence suggest that this axis may exist either in CD4 + FoxP3 + T cells or CD8 + T cells. This pathway could lead T cells to strong antitumor cytotoxic activity in a tumor-specific manner. Furthermore, CD137 is one of the most expected targets for the development of agonist immunotherapies. The identification of CD137 + Eomes + FoxP3+/- T cells could be a key element in the selective activation of the most anti-tumor cells in the HPV + OSC microenvironment.

## Introduction

The incidence of human papillomavirus-related squamous cell carcinoma of the oropharynx (HPV + OSC) has been increasing over the past 30 years. Chronic HPV infection is supplanting alcohol and tobacco intoxications as the leading cause of oropharyngeal cancer in developed countries (Rettig et al. 2018). Patients with HPV + OSC enjoy better survival and respond better to radiotherapy and chemotherapy. Prognostic disparities exist, poorly explained by clinical features (Ang et al. 2010).

The HPV + OSC microenvironment is richer in CD4 + and CD8 + lymphocytes (Badoual et al. 2013; Outh-Gauer et al. 2018) defining an immunologically “warm” microenvironment compared to non-Human Papillomavirus related oropharyngeal squamous cell carcinomas (HPVneg-OSC) (Wang et al. 2019). The antitumor action of cytotoxic CD8 + T cells underlies the efficacy of PD-1/PD-L1 immune checkpoint blockers (Kumagai et al. 2020). Regulatory T cells (T_REG_) are mainly described as immunosuppressive and protumoral in most solid cancers (Aksoylar and Boussiotis 2020).

However, T_REG_ are paradoxically associated with a better prognosis in HPV + OSCs (Cécile Badoual et al. 2006; Cioni et al. 2019; Hur et al. 2022; Liu et al. 2022).

The aim of this paper is to present the following medical hypothesis: CD137 and Eomes constitute a specific T cell activating axis, which results in the reprogramming of T_REG_ into effector CD4 + FoxP3 + T cells and the tumor-specific activation of CD8 + T cells. Long HPV infection and the existence of HPV specific T cells oriented in an effector phenotype might explain favorable prognosis in HPV + OSCs.

## Hypothesis

### Existence of effector CD4 + FoxP3 + T cells that are not T_REG_

The transcription factor FoxP3 is the basis for the identification of T_REG_ (Hori et al. 2003). Among CD4 + FoxP3 + T cells, some have effector functions (Atif et al. 2023).

FoxP3 appears insufficient to explain the maintenance of a constant T regulatory phenotype and function (Ohkura et al. 2012). Variation in this regulatory/effective function of CD4 + CD25 + FoxP3 + T cells is a consequence of TCR engagement, epigenetic modifications (methylation profile, histone modifications, microRNA) and the influence of the microenvironment. TCR engagement and epigenetic modifications on FoxP3 expression have been shown to be complementary and independent events (Miyara et al. 2009).

The involvement of the regulatory transcription factor Eomesodermin (Eomes) orienting T_REG_ into effector CD4 + FoxP3 + T cells has been reported. These CD4 + FoxP3 + Eomes + T cells also express Granzyme B, a marker of cytotoxicity, and are able to clear the tumor in a CD8 + T cell-deprived mouse model (Akhmetzyanova et al. 2016).

On the contrary, Blimp1 transcription factor (PRDI-BF1, gene: PRDM1), an IFN-β gene repressor, could have an opposite role to Eomes transcription factor. Dixon et al. found high Blimp1 + T_REG_ levels in tumors and experiments of deleting Blimp1 in T_REG_ drove them in effector CD4 + T cells as Eomes does (Dixon et al. 2021).

### CD137 activating receptor

CD137 (4.1BB - gene: TNFRSF9) has long been described as one of the most activating receptors in CD4 + and CD8 + T cells since its discovery in 1992 (Uchida et al. 1992). At the time of its discovery, CD137/4.1BB was identified as a discriminator between active and quiescent T cells (Pollok et al. 1993). CD137 receptor belongs to the Tumor Necrosing Factor (TNF) Receptor superfamily. Its ligand (CD137-L/4.1BB-L) has remained unique since its discovery in 1993 (Goodwin et al. 1993).

In addition to its direct activating impact through binding with its ligand, unbound CD137-Ligand (4.1BBL - gene: TNFSF9) is suggested to positively influence immunosurveillance. This involves the activation of CD103 + dendritic cells that secrete IL-12, a crucial interleukin for immunosurveillance (Zitvogel and Kroemer 2014), and the preservation of the antitumor M1 (CD163-) polarization of macrophages (Kang et al. 2017).

Links with PD-1 expression on effector T cells in a self-regulating balance to maintain the homeostasis of T cells function and population have been described and made CD137 a perfect candidate for costimulation immunotherapies (Chen et al. 2015).

A CD137+/Eomes + axis has been described in several publications (Dhume et al. 2022; Lupar et al. 2015; Mittal et al. 2018).

### T_REG_ reprogramming in effector CD4 + FoxP3 + T cells via CD137 and Eomes

The involvement of Eomes orienting T_REG_ into the effector FoxP3 + may depend on TCR/MHC activation through CD137. Several studies have reproduced this polarization using anti-CD137 agonistic antibodies (Chen et al. 2015; Lupar et al. 2015; Akhmetzyanova et al. 2016; Atif et al. 2023). The cytotoxicity indicator protein Killer cell lectin-like receptor subfamily G member 1 (KLRG1) has also been identified and associated with Eomes as a marker of an activated T population in CD4 + and CD8 + cells (Curran et al. 2013; Hu et al. 2020). The inhibitory action of CD137 on the conversion of conventional CD4 + to T_REG_ has been demonstrated via IFN-γ production (Madireddi et al. 2012). CD137 has been shown to activate dendritic cells and NK cells (Houot and Kohrt 2014; Knitz et al. 2021).

CD137/Eomes could be an axis dedicated to the reprogramming of T_REG_ into effector CD4 + FoxP3 + T cells.

A retrocontrol mechanism exists. Following the activation of CD137, a mechanism of “de-activation” is set up and a memory profile is generated (decrease in the expression of Eomes and Tbet and of the inhibition proteins TIM3, LAG3, CTLA4 and increase in the memory-related transcription factors KLF6, JUN, JUN6) (Long et al. 2015).

### CD137/Eomes axis in CD8 + T cells

CD8 + T cells are the main supporting cells in the fight against squamous cell carcinoma (Woolaver et al. 2021). There is less evidence for a cytotoxic role of a CD137/Eomes axis in CD8 + T cells (Hu et al. 2020; Knitz et al. 2021) despite an early publication (Pearce et al. 2003). An CD137/Eomes axis should be sought in order to overcome contradictory data about CD8 + CD137 + specificity.

Antitumor specificity after CD137 activation has been described for CD8 + T cells (Gros et al. 2014; Ye et al. 2014). Activated CD137 induces the transmission of an activation message to the NFkB pathway via its TRAF1-associated transmembrane domain. TRAF1-mediated activation of NFkB occurs via an alternative pathway through NIK (NFkB inducing kinase) and follows TCR engagement. Yet, increased phosphoinositide 3-kinase (PI3K), part of the signaling cascade induced by TCR activation (So and Fruman 2012), is found among CD8+ (and CD4+) T cells populations expressing Eomes+ (Knitz et al. 2021). A distinction is made between activated “tumor-reactive” T cells and “bystander T cells”. This question also concerns the effect, specific to a tumor epitope or “bystander” effect (Joshi et al. 2019). Tumor specificity could be defined with the expression of CD137 as a surrogate marker, which is associated with a specific, prolonged and CD28-independent T cell response (Geuijen et al. 2021; Hu et al. 2020).

Memory resident CD8 + T cells (T_RM_) have recently been identified as a population of non-circulating, tissue-persistent cytotoxic T cells that present themselves to malignant cells in solid tumors. These cells are defined by the expression of the integrins CD103 (which bind to E-Cadherin of tumors and epithelial regions) and CD49a and the C-type lectin CD69 and by a profile of transcription factors: Runx3+, Notch+, Hobit+, Blimp1+, BATF+, AHR+, Eomes- and Tbetlow (Mami-Chouaib et al. 2018).

T_RM_ migrate to head and neck tissue thanks to the CXCR6-CXCL16 axis (Karaki et al. 2021). T_RM_ express significant levels of Granzyme B, IFNγ and TNFα involved in cytotoxicity. Their presence is associated with a better prognosis, which has also been found among HPV-OPSCC (Hewavisenti et al. 2020; Solomon et al. 2019; Welters et al. 2018). CD103 expression by CD8 + T cells has been shown, along with CD39, to be an identifying marker for tumor-reactive CD8 + T cells in solid cancers (Duhen et al. 2021).

Notably, CD137 has been shown to play a role in T_RM_ differentiation in an antigen-specific manner, paradoxically increasing T_RM_ population without mTOR or TRAF1, suggesting the existence of an alternative signalization axis (Zhou et al. 2017, 2019).

The lack of Eomes expression seems to be in contradiction with the previously cited data, a contradiction that could be explained by the existence of variation over time of these elements and the existence of a retrocontrol mechanism. In CD8 + T cells, the decrease of Eomes corresponds to the increase of CD226 (Weulersse et al. 2020).

### Relation with HPV-driven cancers

CD137/Eomes T-cells axis both in effector memory CD8 + T cells and FoxP3 regulatory T cells has never been studied in HPV + OSCs. This immunologically “warm” cancer, caused by chronic infection, has a particular specificity profile (Zhou et al. 2017). It also has the particularity of having highlighted a favorable prognostic role of CD4 + FoxP3 + T lymphocytes (Cioni et al. 2019; Hur et al. 2022; Liu et al. 2022).

HPV is a non-enveloped circular double-stranded DNA virus. HPV infections are the leading cause of sexually transmitted diseases worldwide. Certain HPV subtypes, 16 and 18, present a high risk of cancerous transformation. Subtype 16 is predominant in head and neck carcinomas (Castellsagué et al. 2016). Oncogenic HPVs cause genetic changes responsible for tumor apparition, first described in 1976 (zur Hausen 1976). In 1983, Kari Syrjänen was the man who first suspected HPV involvement in Head and Neck cancers (Syrjänen et al. 1983). Tumorigenesis is linked to the production in the infected cells of E6 and E7 viral onco-proteins as E6 and E7 respectively alter p53 and the retinoblastoma pocket protein (pRB) leading to unregulated proliferation and immortality (Leemans et al. 2018).

Contrarily to alcohol and tobacco-driven cancers, and to HPV-driven cervical cancers, HPV + OSCs, evidence for dysplastic precursor lesions is very scarce (Leemans et al. 2018). HPV + OSCs have been explained as the consequence of a discontinuous basal membrane inside tonsillar crypts (Smola 2017). Lower rate of HPV chronic infection transformation in cancer in the oral cavity compared to the oropharynx (3.9% vs. 47% according to Castellsagué et al.) advocate for a specific matter of immune control in the tonsillar tissue (Castellsagué et al. 2016; Leemans et al. 2018).

Chronic HPV infection precedes the onset of cancer by many years. This chronic infection may potentiate a specific effector immune state through the chronic and long-standing expression of CD137. Subsequently, when the immune system’s cleaning capacities are surpassed (Hanahan and Weinberg 2011), cancer manifests as a clinically detectable disease. The polarization of a greater number of CD4 + T cells into effector cells and their paradoxically favorable role in comparison with HPVneg-OSCs might be a stigma of this pre-cancerous state.

HPV-specific T cells have been demonstrated in a broad repertoire in cervical cancers (de Vos van Steenwijk et al. 2010). Above all, CD137 has been predominantly found in HPV-specific CD4 + and CD8 + T cells (de Vos van Steenwijk et al. 2010). In a mouse model of virus-induced tumors, specific antigenic stimulation by TCR engagement was all the stronger the more viral antigens were expressed by tumor cells (Akhmetzyanova et al. 2016). CD137/Eomes axis and the presence of HPV-specific T cells is worth exploring to explain the favorable prognosis associated with HPV + OSCs compared with HPVneg-OSCs.

### Perspectives

**Fig. 1:**
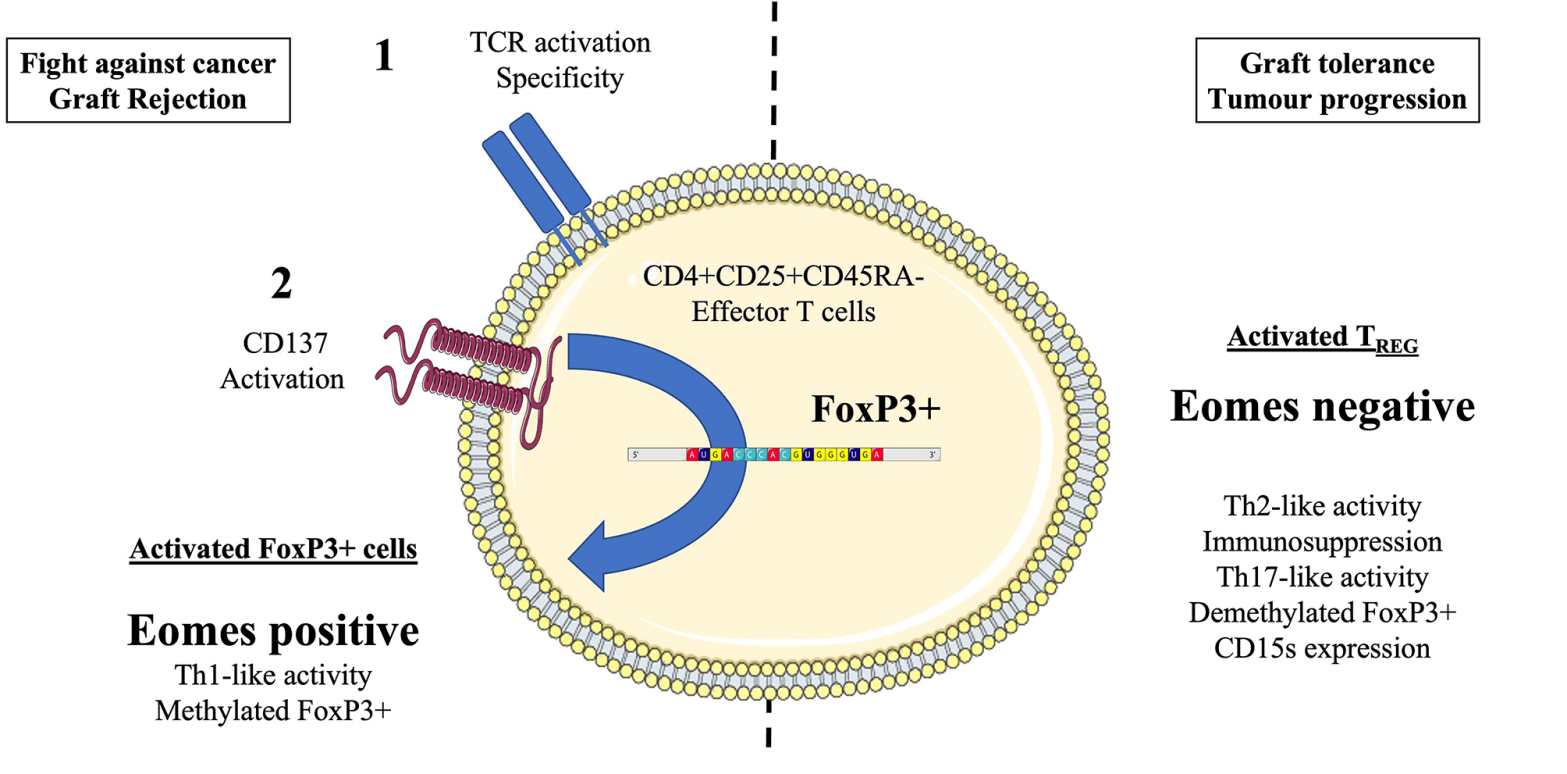
Medical hypothesis of a TCR-dependant activation axis via CD137 and transcription factor Eomesodermin (Eomes) in CD4 + FoxP3 + T cells. The Figure was partly generated using Servier Medical Art®, provided by Servier, licensed under a Creative Commons Attribution 3.0 imported license

The identification of this axis and the proof of its activated cytotoxic and tumor-specific action would concern both the field of cancer diseases and solid organ transplantation (Fig. 1).

## Evaluation of the hypothesis

### Study of function

The first objective would be to determine whether there is a CD137/Eomes axis activating cytotoxic T cells, and polarizing T_REG_ into FoxP3 + effector cells, in HPV + OSCs, and compared to HPVneg-OSCs.

A readily achievable initial approach is to confirm the existence of T cells expressing both CD137 and Eomes and/or FoxP3 + within the HPV + OSC microenvironment using in situ analysis of tumor tissue using immunofluorescence (IF) or immunohistochemistry (IHC). These analyses can provide a quantitative evaluation in terms of the number of relevant cells per mm2 and allow for a comparative assessment with HPVneg-OSCs samples. The identification of cells and their relationships to each other within the microenvironment could be specified on patient tissues via spatial single-cell RNA-sequencing or flow cytometry by time-of-flight. The activated profile of the cells would be enhanced by studying the methylation of genes coding for transcription factors such as FoxP3 + and Eomes by genome-wide analysis in relation to the expression of CD137/Eomes by T cells (CpG methylation analysis by bisulfite sequencing) (Dhume et al. 2022). The markers and transcription factors studied correspond to a function or its regulation: activated Treg (CD4 + CD45RA-FoxP3 + CD15s+), Effectors CD4 + FoxP3 + T cells (CD4 + CD45RA-FoxP3 + Eomes+).

### Study of specificity

In the context of HPV + OSCs, the influence of the presence of viral proteins and their production by infected cells on this specificity should be determined in HPV + OSCs and compared to HPVneg-OSCs.

TCR sequencing techniques - bulk sequencing of pooled lymphocytes, from FFPE input and TCR clonality metrics (CDR3 sequencing) would be required or single-cell sequencing techniques of VDJ coding sequences for T cell diversity.

### Study of the temporal evolution of T cell activation

The third step would aim to explain the contradictions detailed above by looking for the existence of a temporality in the profile and function of T cells after activation of the TCR/CD137/Eomes axis according to the following hypothesis: 1/ an activated phase (CD137+, Eomes+), 2/ a depleted phase (Eomes decreases, CD226 + is expressed) and then inhibited (PD-1+, CTLA-4+ / TIM-3+) and, 3/ a phase of constitution of a resident memory population (CD103+, KLF6+, JUN+, JUN6+).

A murine model could prove valuable during this stage of hypothesis evaluation and would unfold as follows. Two mouse populations would be utilized: a first population of highly immunogenic Balb/c mice (Akhmetzyanova et al. 2016), and a second population of RAG-1 (Recombination-activating gene-1) double-negative mice (Knitz et al. 2021) corresponding to functional inactivation of T cells. Each population would be transfected with a murine retrovirus-induced tumor cell line of C57BL/6 mouse origin, specifically FBL-3 cells (Glynn et al. 1968), selected for their resemblance to HPV + OSCs due to tumorigenesis induced by an oncogenic virus. At a designated time, approximately around day 10 from tumor transfection, mice from both populations would receive a CD137 agonist in the form of a monoclonal antibody. Individuals would be regularly sacrificed before and after tumor transfection. Tumor growth and therapy response would be comparatively assessed between each population, along with the composition of the tumor microenvironment, through in situ analysis using IHC or IF, or non in situ through RNAsequencing or flow cytometry. The differential presence of CD137+/Eomes + T cells and their evolving profiles would be evaluated between the two populations. Comparison with RAG-1 -/- mice will help us determine the contribution of TCR engagement and antitumor specificity in the activation process. An additional analysis opportunity would be provided by depleting CD8 + or CD4 + T cells in the Balb/c population before CD137 activator treatment to discern the influence of CD8 + and CD4 + populations on Eomes expression.

Single-cell phenotypic characterisation of T cells and the microenvironment, in situ or not, by RNAsequencing and flow cytometry would allow to study the hypothesis of a narrow control of Eomes, signaled by the Eomes-dependent increase of CD226+.

Markers of lymphocytes depletion after activation would be studied such as PD-1+, CTLA-4 + and TIM-3 + for CD4 + T cells. Markers of memory residence and function would be studied: CD103+, KLF6+, JUN+, JUN6+.

## Conclusion

The hypothesis of a highly cytotoxic activating population among T cells exists, even under the cover of markers previously identified as regulatory or immunosuppressive. The identification of an activation pathway specific to these effector cells via CD137 and the transcription factor Eomes after TCR engagement offers the opportunity to target a tumor-specific population with a strong cytotoxic activity.

Such a discovery offers tempting prospects in cancer therapy. Immune checkpoint inhibitors are indicated in clinical practice in head and neck cancer with a significant response rate (Burtness et al. 2019). However, a significant proportion of patients do not respond or show progression with checkpoint inhibitors (Kang et al. 2022). CD137 agonists exist and could be combined as synchronous or delayed systemic combination therapy. Animals’ studies showed promising results and several human assays have been carried out (Chu et al. 2019).

However, some data remain contradictory, such as the accumulation of polyclonal T cells in the liver under anti-CD137 agonists, with no benefit for clearance of transplanted tumors in this organ. These applications could concern all other solid cancers and also have implications for organ transplantation (Dubrot et al. 2010).

The immunological treatment of cancers is the revolution of the 21st century. The personalization of treatments based on an understanding of the tumor microenvironment is the holy grail of clinical and basic translational research. The hypothesis of new axes, specific to antitumor cytotoxicity, deserves to be explored.

## Data Availability

Not applicable.

## Abbreviations

HPV: Human papillomavirus
HPV+OSC: Human papillomavirus-related squamous cell carcinoma of the oropharynx
HPVneg-OSC: Non-Human Papillomavirus related oropharyngeal squamous cell carcinomas
PD-1: Program-Death-1
PD-L1: Program-Death-Ligand-1
CD: Cluster of differentiation
TREG: Regulatory T cells
TRM: Memory resident CD8 + T cells
TNF: Tumor Necrosing Factor
MHC: Major Histocompatibility complex
IL: Interleukine
Eomes: Eomesodermin transcription factor
KLRG1: Killer cell lectin-like receptor subfamily G member 1
pRB: Retinoblastoma pocket protein
FFPE: Formalin fixed paraffin embedded tissue
